# Hybrid Particle Swarm Optimization and Its Application to Multimodal 3D Medical Image Registration

**DOI:** 10.1155/2012/561406

**Published:** 2012-08-22

**Authors:** Chen-Lun Lin, Aya Mimori, Yen-Wei Chen

**Affiliations:** College of Information and Science, Ritsumeikan University, Shiga 525-8577, Japan

## Abstract

In the area of medical image analysis, 3D multimodality image registration is an important issue. In the processing of registration, an optimization approach has been applied to estimate the transformation of the reference image and target image. Some local optimization techniques are frequently used, such as the gradient descent method. However, these methods need a good initial value in order to avoid the local resolution. In this paper, we present a new improved global optimization approach named hybrid particle swarm optimization (HPSO) for medical image registration, which includes two concepts of genetic algorithms—subpopulation and crossover.

## 1. Introduction

 In the area of medical image analysis, multimodality 3D image registration is an important issue [1]. The purpose of image registration is to register a target image (moving image) to a reference image (fixed image) so that we can combine the information of two images to obtain more detailed information or some specific features. For example, the PET image usually shows metabolic activity of organs and abnormal tissues clearly but lacks the texture of organ tissues. On the other hand, the MR image is described by much complex intensity to represent the texture of organ tissues well. If we implement the MR-PET image registration to combine the information of two images which are different modality, then we can get the accurate shape, volume, and location of abnormal tissues from the registered image. The registration is a very important and helpful preprocessing technique for medical diagnosis or surgical operations. 

 The processing of registration can be seen as an iterated optimization framework, and it can be divided into 3 parts: transformation, cost function, and optimization. In each iteration, the target image is firstly transformed by transformation according to the parameter of the current time. Then, the reference image and the transformed target image are used to calculate the cost function which can evaluate whether the two images are registered or not under the current parameter of transformation. If the images are not registered, an optimization method will be used to adjust the parameters, and a new iteration will start.

 The application of registration can be classified with dimensionality of image, modality of image, and model of transformation. There are 2D to 2D, 3D to 2D, and 3D to 3D image registration for many different application. The 3-D to 3-D image registration usually needs to estimate more parameters than the 2-D to 2-D image registration, so it does require a more advanced optimization method. Then, if two images which have different scope of intensity have to be registered, such as CT-MR registration, it is called multimodal registration. On the other hand, if register two images which have same modality, it is called monomodal registration, such as CT-CT. Depending on the modal of registration, different cost functions have been used, such as the sum of squared intensity difference (SSD) for mono-modal registration or mutual information (MI) for multimodal registration. Moreover, the type of transformation model determines whether a registration belongs to rigid or nonrigid one. If target objects which we want to register are different in shape or deformable such as liver, the nonrigid transformation is used it is called nonrigid registration [2]; otherwise it is called rigid registration. This paper is focused on rigid multimodal 3D medical image registration.

 As our previous explanation, we estimate the parameter of transformation by optimizing a cost function (similarity metric) in the processing of registrations. Some local optimization techniques, such as the gradient descent method, are frequently used for medical image registrations [3, 4]. However, since the transformation parameters are generally nonconvex and irregular, these kind of methods require very good initial values in order to avoid the local minimum.

 To overcome the local resolution problem, the genetic algorithm (GA), which is one of the global optimization techniques, has been proposed for medical image registrations [5]. Although GA is an advanced method for global optimization, it requires huge computation time and lacks the fine tuning capabilities. We need more powerful approach. Particle swarm optimization (PSO) is a new global optimization technique. This method is a stochastic, population-based evolutionary computer algorithm [6, 7]. PSO is an extremely simple algorithm, and it seems to be more effective for optimizing a wide range of functions, and has been shown very effective for 2D rigid image registration [8].

 On the other hand, more transform parameters must be estimated in 3-D image registrations. Our experiments show that conventional GA and PSO cannot find the global optimum well. Thus, we propose a new approach named hybrid particle swarm optimization (HPSO) for PSO. In our proposed method, two concepts of genetic algorithms—subpopulation and crossover—are incorporated into the conventional PSO method to improve the accuracy of that conventional GA and PSO, because these conventional methods can not find the global optimum resolution when we need estimated a huge number of parameters. Experiments are done with both mathematical test functions and medical volume data, and it is demonstrated that the proposed HPSO performs much better results than conventional gradient decent, GA, and PSO methods.

 The paper is organized as follows: the image registration technique is summarized in Section 2, the particle swarm optimization (PSO) and our proposed hybrid particle swarm optimization (HPSO) are presented in Section 3, the experimental results with both mathematical test functions and medical volume data are presented in Section 4, and finally the conclusion and future works are given in Section 5.

## 2. 3D Image Registration

**Figure 1:**
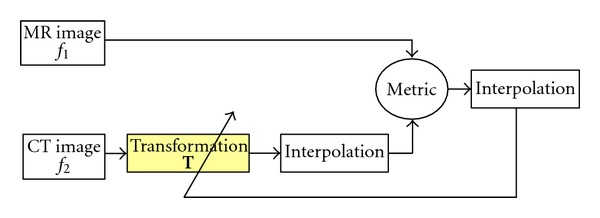
Framework of medical image registration.

 Medical Image registration is one of the fundamental tasks within medical image processing. The framework of medical image registration is shown in Figure 1. Two volumes of medical data that have to be registered are given as the fixed image and the moving image. The fixed image is denoted by *f*_1_(**x**), where **x** is a set of coordinates. The moving image is similarly denoted by *f*_2_(**x**). Given **T** is a transformation from the coordinate frame of the fixed image to the moving image, *f*_2_(**T**(**x**)) is the moving image associated with fixed image *f*_1_(**x**). In order to simplify some of the subsequent equations, we will use **T** to denote both the transformation and its parameterization. Here, we used an estimation of the transformation that registers the fixed image and moving image by maximizing their cost function (similarity metric) as shown in (1):

(1) $$\begin{array}{c} \hat{T}=\arg\underset{T}{\max}\;\text{Mtric}\left[f_{1}\left(x\right),f_{2}\left(T\left(x\right)\right)\right], \end{array}$$

where **x** is the coordinate of a 3D or 2D point.

### 2.1. Transformation

 Registration can be seen as a process of finding the spatial transformation that maps points from one image to the corresponding points in another image. Here, we focus on rigid 3-D global transformation. The rigid transformation deals with 6 degrees of freedom for 3-D object translation and rotation. The rigid transform for 3-D object can be expressed by (2):

(2) $$\begin{array}{c} T_{\text{Global}}\left(x\right)=Rx+t=\left(\begin{array}{ccc} \cos\beta \cos\gamma & \cos\alpha \sin\gamma +\sin\alpha \sin\beta \cos\gamma & \sin\alpha \sin\gamma -\cos\alpha \sin\beta \cos\gamma \\ -\cos\beta \sin\gamma & \cos\alpha \cos\gamma -\sin\alpha \sin\beta \sin\gamma & \sin\alpha \cos\gamma -\cos\alpha \sin\beta \sin\gamma \\ \sin\beta & -\sin\alpha \cos\beta & \cos\alpha \cos\beta \end{array}\right)\left(\begin{array}{c} x\\ y\\ z \end{array}\right)+\left(\begin{array}{c} t_{x}\\ t_{y}\\ t_{z} \end{array}\right), \end{array}$$

where *α*, *β*, *γ* are rotation angles around each axis and *t*_*x*_, *t*_*y*_, *t*_*z*_ are translations around each axis, respectively. It should be noted that there are only two parameters to be estimated for 2D rigid transform.

### 2.2. Metric Function (Mutual Information)

 For multimodality medical image registration, mutual information (MI) is widely used as a similarity metric [9]. MI is an intensity-based similarity measure and is closely related to joint entropy. Given an image *A* and an image *B*, the joint entropy of two images can be calculated by

(3) $$\begin{array}{c} H\left(A,B\right)=-\sum_{a,b}p_{A,B}\left(a,b\right)\log p_{A,B}\left(a,b\right), \end{array}$$

where *p*_*A*,*B*_ is the joint probability distribution function of pixels associated with images *A* and *B*. The joint entropy is minimized when there is a one-to-one mapping between the pixels in *A* and their counterparts in *B*. It increases while the statistical relationship between *A* and *B* weakens. Mutual information can be defined in terms of entropy as follows:

(4) $$\begin{array}{c} MI\left(A,B\right)=H\left(A\right)+H\left(B\right)-H\left(A,B\right), \end{array}$$

where *H*(*A*) and *H*(*B*) are the individual entropies, which can also be represented by the probability distribution function (PDF) as

(5) $$\begin{array}{c} H\left(X\right)=-\sum_{x}p_{X}\left(x\right)\log p_{X}\left(x\right). \end{array}$$

Usually, a discrete joint histogram is adopted to estimate the joint PDF for the calculation of MI. Attempting to find the most complex overlapping regions, we should maximize the individual entropies and minimize the joint entropy which could explain each other well.

## 3. Hybrid Particle Swarm Optimization

 In this paper, we propose a new approach named hybrid particle swarm optimization (HPSO) for 3-D rigid medical volume registration. For describing more details, we show the traditional PSO first.

### 3.1. Particle Swarm Optimization (PSO)

 Assume an extent diffuse population existing which is called a swarm and an individual member of the swarm is termed as a particle. A particle can be imaged as a point in search space. A group of particles tend to cluster at a position where optimized results are identified. Therefore, to achieve particle swarm optimization, each particle adjusts itself by comparing previous experience and its neighbors to obtain the best result. The formula of particle swarm optimization can be represented as below:

(6) vit+1=wtvit+c1·rand·[p_besti−xit]   +c2·rand·[gbest−xit],wt+1=wt+dw; dw=(wmin⁡−wmax⁡)T,xit+1=xit+vit+1.

**Figure 2:**
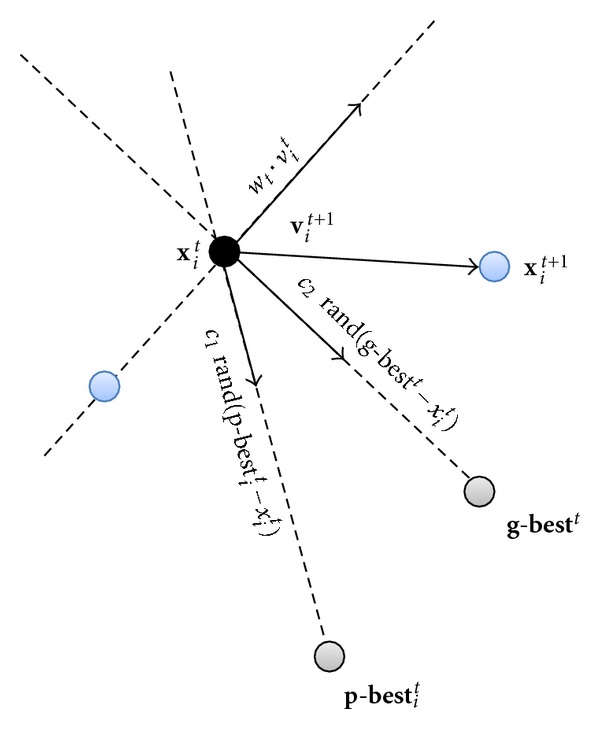
The movement of each particle.

At iteration *t*, **x**_*i*_ is the *i*th particle that moves with a velocity vector **v**_*i*_, **p**_**b** **e** **s** **t**_*i*_ is the personal best of **x**_*i*_, and **g**_**b** **e** **s** **t** is the global best among all particles. *w* means the weight. The initial weight is 0.4. The minimum weight is 0.4 and the maximum is 0.98. *c*_1_ and *c*_2_ represent acceleration constants (*c*_1_ = *c*_2_ = 2.0). rand is a uniformly distributed random number among 0 to 1. The movement of each particle is shown in Figure 2.

**Figure 3:**
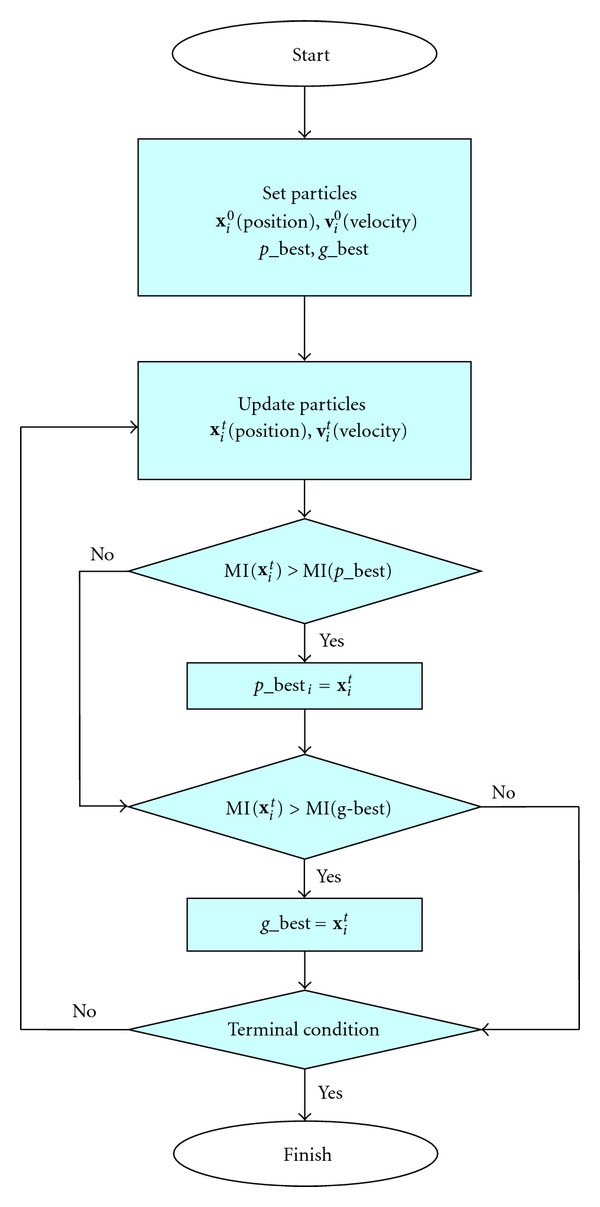
Flowchart of PSO.

The flowchart of PSO for image registration is shown in Figure 3.

In the PSO-based registration approach, the particle **x** is the transform parameter that needs to be estimated, the *p*_best is the maximum MI (cost function) of each particle, and *g*_best is determined by the cluster MI. MI in Figure 2 represents the similarity metric function.

### 3.2. Hybrid Particle Swarm Optimization

**Figure 4:**
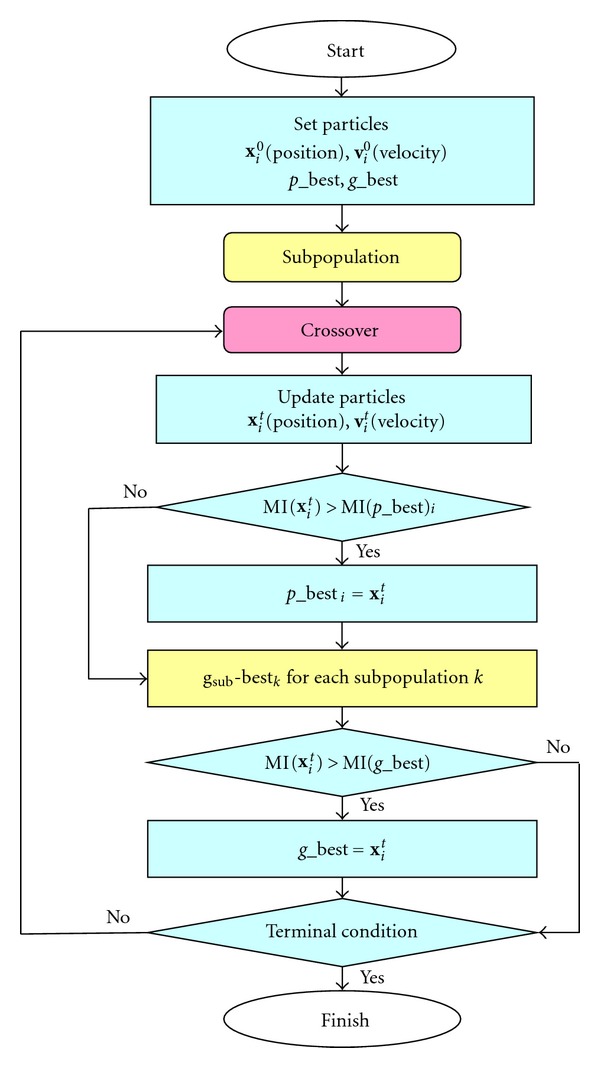
A total of 40 runs of 100 generations each for each function and each method were performed. The population size is 56 for GA, PSO, and HPSO, respectively, and the number of subpopulation is 4 for HPSO. The relative mean square error between the estimated solutions and real solutions is calculated for each trial. Table 1 shows the averaged relative mean square error over 40 trials for each method. Flowchart of HPSO.

 In this section, we propose a new approach named hybrid particle swarm optimization (HPSO) for 3-D rigid medical volume registration. Our method incorporates two concepts of genetic algorithms, which are subpopulation and crossover, into the traditional PSO. We expect that our proposed method will improve the accuracy of registration by taking advantage of subpopulation and crossover. The flowchart of HPSO is shown in Figure 4.

#### 3.2.1. Subpopulation

The particles are divided into a number of subpopulations. Each subpopulation has its own best optimum, *g*_sub_-best_*k*_. The process of PSO is done for each subpopulation group. If the *g*_sub_-best_*k*_ is better than *g*_best, then *g*_best, is replaced by the *g*_sub_-best_*k*_, where *k* is the subpopulation number.

#### 3.2.2. Crossover

The *g*_sub_-best_*i*_ are sorted in order with large mutual information. The top two *g*_sub_-best are selected as parents (**x**_*i*_ and **x**_*j*_) for crossover, where *i* and *j* are their subpopulation number. The offspring are generated for each by arithmetic crossover, which are shown as

(7) xi′=rand·xi+(1−rand)·xj,xj′=rand·xj+(1−rand)·xi,

and the velocities are given by

(8) vi′=viV,vj′=vjV, V=(vi+vj)||vi+vj||,

where rand is a uniformly distributed random number among 0 to 1. The worst particle in the same subpopulation is replaced by the offspring.

## 4. Experimental Results

 In this section, we perform several registration experiments with both test functions and medical volume data to evaluate the performance of the proposed HPSO technique. In the meantime, we also perform conventional GA and PSO for comparison. Finally, we implement the parallel technique to reduce computation cost and show the efficiency of our implementation.

### 4.1. Test Function Evaluation

 In the first part of Section 4, we apply 4 mathematical functions which have different numbers of parameters to estimate the accuracy of our proposed method. The functions which were used are shown as below:

(9) F1:  f(xi ∣ i=1,…,3)=∑i=13xi2, xi∈[−5.12,5.11],

(10) F2:  f(xi ∣ i=1,2)=100(x12−x2)+(1−x12)2, xi∈[−2.048,2.047],

(11) F3:  f(xi ∣ i=1,…,20)=(20×10)+[∑i=120(xi2−10cos⁡(2πxi))], xi∈[−5.12,5.11],

(12) F4:  f(xi ∣ i=1,…,10)=1+∑i=110xi24000  −∏i=110cos⁡(xii), xi∈[−5.12,5.12].

The functions are commonly used for evaluation experiments and cover a variety of characteristics that affect algorithmic performance [10]. *F*1 is unimodal with global minimum at center and has 3 parameters to be estimated. *F*2 is strong epitasis with global minimum at center and has 2 parameters to be estimated. *F*3 is highly multimodal with global minimum at center and has 20 parameters to be estimated. *F*4 is multi-modal with global minimum at corner and has 10 parameters to be estimated. *F*3 and *F*4 are more difficult than *F*1 and *F*2, and *F*3 is the most difficult problem.

As shown in Table 1, both PSO and HPSO can get perfect solutions, and GA can also get a reasonable result for *F*1 and *F*2 in which the number of parameters is only 3 and 2, respectively. On the other hand, increasing the number of parameter (*F*3 and *F*4), both conventional GA and PSO cannot get reasonable results especially for *F*3, while the proposed HPSO can perform much better results than conventional GA and PSO even for *F*3.

**Figure 5:**
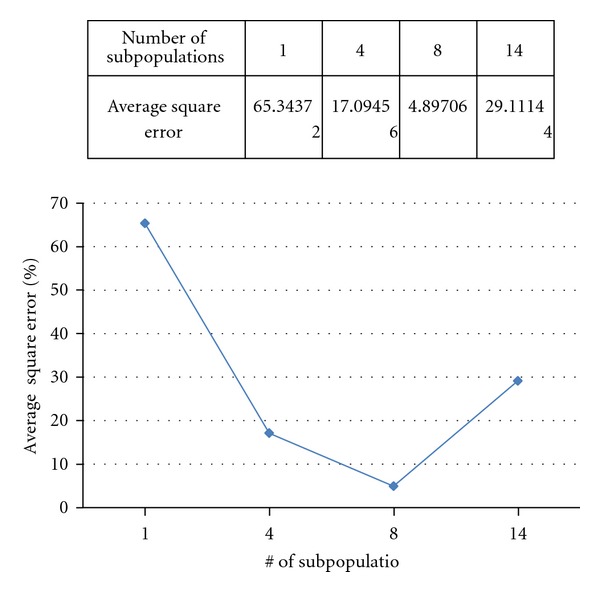
The dependence of optimization accuracy on the number of subpopulations.

According to our experiments, we also found that our method is strongly affected by the number of subpopulations.  Figure 5 shows the dependence of optimization accuracy on the number of subpopulations for *F*3. We change the number of subpopulations (*N*) and perform 40 runs for each *N*. The averaged square error is shown in Figure 5. All of experiments use 5600 particles. We can see that the curve of average square error is getting down when the number of subpopulations (*N*) is increased. The traditional PSO corresponds to *N* = 1. This result indicates that our HPSO method can provide higher accuracy while using more subpopulation. However, if the number of subpopulations is too large, the accuracy will be decreased as shown in Figure 5 because of the limited particle number in a subpopulation.

### 4.2. Medical Volume Data

 In this part, we perform several registration experiments with medical volume phantom data to evaluate the performance of the proposed HPSO technique.

#### 4.2.1. Simulated Data

**Figure 6:**
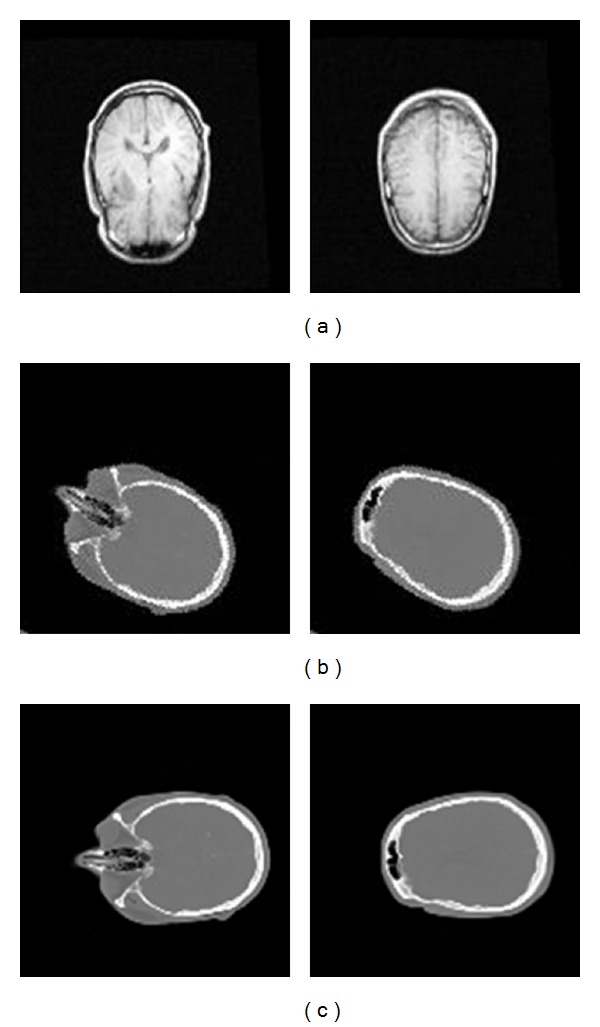
(a) MR volume (fixed image data); (b) CT volume (moving image data) of experiment 1 ((b) to (a)); (c) CT volume (moving image data) of experiment 2 ((c) to (a)).

 We firstly use some simulated data to test our proposed method.  Figure 6 shows some slices of our test data for 2 experiments which have different transformation parameters. The size of each volume is resized to 128 × 128 × 15, and the spacing of each volume is 2.59 × 2.59 × 8.0 (mm).

These simulated data is made by specific parameters which we give it, so that we can easily estimate the result of registration accuracy by using these answers (actual results). Tables 2 and 3 show the comparative results of our HPSO and the previous method (GA and PSO) that diff is defined as difference between experimental results and ground truth.

According to the experimental results which are shown previously, we realize our proposed method can provide more exact result of registration parameters than conventional method. This is the first evidence to prove that our HPSO is a better optimization approach.

#### 4.2.2. Vanderbilt Database

**Figure 7:**
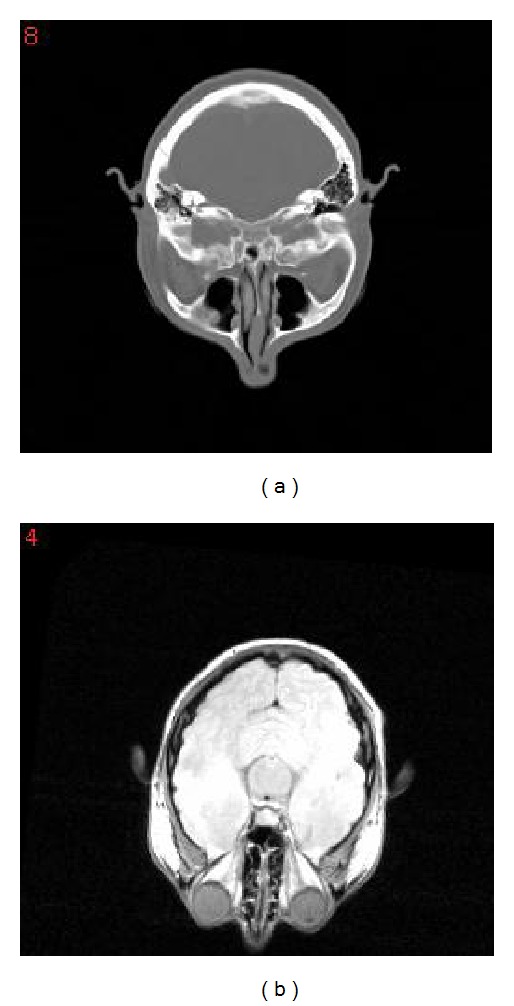
(a) A slice of CT image of Vanderbilt database; (b) a slice of MR image of Vanderbilt database.

 The real medical data (Vanderbilt database [11]) is also used to evaluate the performance of our proposed HPSO (see Figure 7). This database gives both multimodal brain volumes and their marker-based golden standard transforms. These rigid transforms are determined by marker-based prospective registration techniques and represented by eight couples of 3-D points (landmarks) on both of two medical volumes. Such a golden standard transform can be considered as a ground truth (correct one). The size of each volume is resized to 256 × 256 × 29 and the spacing of each volume is 1.25 × 1.25 × 4.0 (mm).

Here we try gradient decent method, GA, PSO, and HPSO on CT to MR registration problem. A total of 3 runs with different random initial values were performed. Eight landmarks in the volume of Vanderbilt database are used for quantitative evaluation. Equation (12) is a measure used to compare the registration accuracy between experiment-obtained transform (*g*) and the golden standard transform (*g*_Golden_). **x**_*i*_  (*i* = 1,2,…8) is coordinate of the landmark *i*:

(13) $$\begin{array}{c} e=\frac{1}{8}\sum_{i=1}^{8}||g\left(x_{i}\right)-g_{\text{Golden}}\left(x_{i}\right)||. \end{array}$$

The averaged accuracy of registration results for each method is shown in Table 4. It can be seen that our proposed HPSO performs much better results than conventional gradient decent method, GA and PSO.

### 4.3. Parallel Implementation

 The efficiency of registration is also an important issue as well as registration accuracy [12, 13]. Although we have shown the accuracy improvement in the previous sections, almost global optimization has a big disadvantage: a huge computation cost. We applied our proposed HPSO to a computer which has 4-core CPU and implement it with parallel technique to overcome this problem. For registering two images with size of 256 × 256 × 29, the average computation cost is reduced to 1893.637 sec from 3661.747 sec by our implementation. These results indicated that the computation cost can be significantly reduced by parallel implementation.

## 5. Conclusion and Future Works

 This paper introduces a new global optimization approach named hybrid particle swarm optimization (HPSO) which incorporates two concepts: subpopulation and crossover of genetic algorithms into the conventional PSO. We performed both functional evaluation and 3-D rigid medical volume registration to estimate the proposed method. First, 4 functions have been applied to test the ability of our method for finding the global resolution and avoiding the local resolutions. Then, in case of 3-D rigid medical volume registration, we applied our HPSO to both simulated data and real medical data (Vanderbilt database) to discover the capability of this method for real medical volume registration. In order to compare conventional methods such as GA and PSO are also used for each experiment. All experimental results prove that the proposed HPSO performs much better results than conventional GA and PSO. Therefore, we can summarize that our HPSO is an advanced optimization method. The large computation cost can be significantly reduced by parallel implementation.

 Furthermore, this work can be extended to 3D nonrigid registration in order to cover more computer-assisted surgery application such as real time liver tumor resection.

## Figures and Tables

**Table 1:** Comparison results of test functions.

	Number of parameters	GA (%)	PSP (%)	HPSO (%)
*F*1	3	0.09	0	0
*F*2	2	0.89	0	0
*F*3	20	70.13	67.65	11.49
*F*4	10	15.62	11.38	1.64

**Table 2:** Comparison result of registration parameters ((b) to (a)).

	Translation				Rotation (degree)			
	*T*_*x*_	*T*_*y*_	*T*_*z*_	diff	*θ*_*x*_	*θ*_*y*_	*θ*_*z*_	diff
Answer	20.0	10.0	2.0	—	0.0	0.0	120.0	—
GA	−2.6	−13.3	−0.01	10.84	44.93	45.25	149.49	23.42
PSO	−4.84	−9.95	0.09	10.64	53.41	60.39	120.27	26.87
HPSO	20.04	11.16	1.75	0.40	−0.25	−0.48	117.57	0.83

**Table 3:** Comparison result of registration parameters ((c) to (a)).

	Translation				Rotation (degree)			
	*T*_*x*_	*T*_*y*_	*T*_*z*_	diff	*θ*_*x*_	*θ*_*y*_	*θ*_*z*_	diff
Answer	10.0	5.0	2.0	—	0.0	0.0	90.0	—
GA	5.33	0.21	0.50	2.29	31.58	40.42	61.94	19.49
PSO	−4.84	2.25	−0.03	2.05	29.21	35.67	92.30	15.39
HPSO	9.98	5.11	1.83	0.07	−0.20	−0.52	87.95	0.71

**Table 4:** Comparison of registration accuracy (mm).

Gradient decent	GA	PSO	HPSO
5.62	9.75	8.89	2.36
